# Construction and Application Research of the Visual Image Obstacle Type Recognition Model Based on the Computer-Expanded Convolutional Neural Network

**DOI:** 10.1155/2022/3123448

**Published:** 2022-09-21

**Authors:** Yuchen Xian

**Affiliations:** School of Software Technology, Dalian University of Technology, Dalian 116000, Liaoning, China

## Abstract

Due to the development of computer vision technology and image processing technology, obstacle recognition technology has been widely used in military and scientific research fields. However, most of the existing image-based recognition technologies are easily affected by environmental factors, which makes the application scenario of this system more fixed and cannot be applied in complex environments. This paper mainly focuses on the traditional obstacle detection and type recognition method recognition accuracy, reliability and universality is difficult to meet the technical requirements of intelligent vehicles and unmanned vehicles, traditional detection equipment cost is expensive, and other problems. There are many traditional obstacle detection methods, which basically start from the color, edge, and other information of the target object to do detection and recognition research, but their recognition accuracy, reliability, and universality are difficult to meet the technical requirements of intelligent vehicles and unmanned vehicles, and the detection equipment is expensive. The dilated convolutional neural network has the ability to learn autonomously, using the original image as input, without the cumbersome preprocessing process and can extract features of the target object one by one to achieve more accurate recognition. This design will be based on the expanded convolutional neural network, design an obstacle type detection and obstacle recognition application with high recognition accuracy, and good generalization, in which this paper applies the hierarchical structure of the expanded convolutional neural network weight sharing to learn the characteristics of various types of obstacles and extract the global features with characterization significance, combined with the ROI algorithm to achieve real-time obstacle detection and high accuracy type recognition. The ROI algorithm is combined to achieve real-time obstacle detection and high-precision type recognition.

## 1. Introduction

In recent years, self-driving cars and autonomous mobile robots have received increasing attention from science and technology, and obstacle detection and recognition is the key to their development [1].

For example, in the military, it can replace personnel for reconnaissance, patrol, search, rescue, and other tasks to reduce unnecessary casualties on the battlefield; in civil use, it can realize automatic obstacle avoidance and unmanned driving of cars and also serve as daily traffic identification and navigation for people with visual impairment [2]; in scientific research, this technology can also be used for high-risk exploration work such as deep sea and space exploration, and for various scientific experiments to collect important information. It is foreseeable that on the basis of driverless vehicles, autonomous mobile robots and a variety of autonomous navigation systems, significant changes will take place in the future. But even so, it is a difficult technical challenge to solve [3].

**Figure 1:**
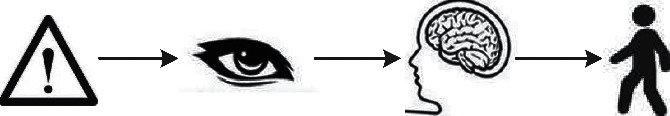
Diagram of the human obstacle avoidance process based on visual perception.

Obstacle recognition technology is the advanced technology and research results of computer vision, pattern recognition, machine learning, artificial intelligence, and other multidisciplinary and multidisciplinary fields, which is the inevitable result of technological development. Humans can identify and avoid obstacles through vision, brain, and body movements during travel. Firstly, visualization methods are used to obtain traffic conditions in the direction of vehicle travel; on this basis, the brain identifies and judges the obstacles in the line of sight through the traffic information transmitted by vision and feeds them back to the limbs; in order to achieve obstacle avoidance, the limbs will make different tendency avoidance movements according to the signals from the brain (see Figure 1).

So-called obstacle recognition is the use of computers or other computing processors and image acquisition devices to simulate the automatic recognition and feedback of any obstacle by the human brain and eyes. The recognition system has two basic elements: one is the machine vision (camera or other image capture device) and the other is the computing processor (similar to the human brain). If control elements are added, functions such as obstacle avoidance for human bodies, such as cars and automatic mobile robots, can be implemented, which are based on detection, recognition, and control. How to improve the adaptability and real-time performance of obstacles in complex environments has received increasing attention from researchers [4].

We know that 80% of human information is obtained from vision and 20% is obtained through hearing. At the same time, according to relevant studies, about one-fifth of the human population is active in image processing, so vision is very important when driving a car. In addition, machine vision-based environment perception technology is a nonintrusive method of information acquisition, which can achieve zero pollution of the road environment. Therefore, the development of vision-based obstacle detection and recognition technology will be a pioneer in the field of intelligent control of automobiles [5].

Extended convolutional neural network is an important branch in current computer vision research, which has a wide range of applications in image classification, target detection, target tracking, pose estimation, motion recognition, and scene labeling. Obstacle detection and recognition are object detection and tracking, while object distance and movement direction are estimates of object behavior and pose, and image classification and scene labeling can classify and label images. In conclusion, the extended convolutional neural network is an effective method for obstacle detection and recognition. In image recognition, the widely used convolutional neural network with multi-level feature extraction is used to detect and recognize obstacles more accurately [6].

## 2. Introduction to Related Technologies

### 2.1. Convolutional Neural Network Theory

There are many traditional obstacle detection methods, which mainly focus on object color, edge, and other information for recognition and identification, but it is difficult to meet the technical requirements of automobiles and autonomous vehicles due to recognition accuracy, reliability, and universality. In contrast, convolutional neural network is an automatic learning method, which does not need to go through complicated preprocessing and can extract the features of the target step by step, thus achieving higher accuracy. Also, the method requires only a simple test device, such as a PC and an on-board computer [7].

**Figure 2:**
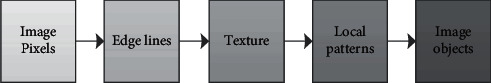
Hierarchical structure of image features.

**Figure 3:**
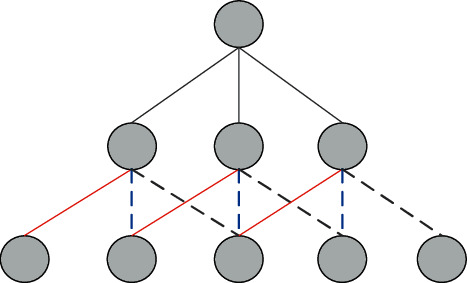
Weight sharing.

A video capture program can be used. Compared with traditional detection methods, convolutional neural networks have the following advantages :

1. Hierarchical structure. The hierarchical structure of the convolutional neural network is designed to match the hierarchical distribution of image features.
2. The hierarchical structure of the convolutional neural network is designed to fit well with the hierarchical distribution of image features. The hierarchical structure of image features is shown in Figure 2.
3. In adjacent regions, a boundary line is formed by pixel information from multiple regions, and each line along different directions is combined to form a texture of an image, while a local image consists of multiple locations or key points, and the target object can be reproduced by the combination of multiple local graphics. Convolutional neural network is a layered structure consisting of convolutional and pooling layers, which can extract the image in layers and describe the image object in a holistic form [8].
4. Connectivity. Based on the neighborhood feature similarity of the image itself, the convolutional neural network utilizes local connectivity to perform large-scale sparse of the network. Neighborhood feature similarity can also be understood as the stability of neighborhood features, i.e. [9], the statistical properties of a subregion in the original image have similar statistical properties to several of its neighboring subregions. Convolutional neural networks learn the characteristics of an image by means of local connectivity, which is achieved by combining regions with similar properties, replacing the similar properties of the whole region with one typical feature, and finally combining all the different features to form a complete target object [10].
5. Using the local connection method can effectively reduce the training parameters of the network. On the basis of 1000*∗*1000, the number of neurons in the next hidden layer is 1000000, and the training parameters of the model are 1000*∗*1000*∗*1000000 = 10^12 when fully connected; however, when local connections are made between neurons, 100000 hidden layer neurons are connected with 10*∗*10 local regions, the network parameters become 10*∗*10*∗*1000000 = 10^8, which is a reduction of 4 orders of magnitude in local connectivity compared to full connectivity and has great advantages in practical applications. Especially in larger samples, the learning efficiency of the convolutional neural network is significantly improved compared with the fully connected case [11].
6. Weight sharing. CNN has a better feature than other neural networks, i.e., weight sharing, and the structure of weight sharing is given. Convolutional kernels of the same type, whose weights and offsets are shared, act on the original image in a series of covariates in sequence and finally obtain specific types of image features. Using multiple convolutional kernels, multiple feature sets can be obtained, and multiple feature sets are combined to form a complete image [12]. Weight sharing and local linking have similar functions, both significantly reduce the complexity of the network and are effective in reducing computation and accelerating learning (see Figure 3).

### 2.2. Dilated Convolution in Computer Vision

Dilated convolution is a kind of “ATrry convolution” which has been developed into wavelets. In comparison with pure convolutional networks, a new approach is proposed, namely, expanded convolution, which expands the network's receptive area while adding some linear parameters. Pooling operation is another method to increase the field of view (FOV) of the network in an exponential way; however, it loses much background information and requires other operations [13], such as deconvolution or up-sampling, to obtain the same input size. In contrast, extended convolution not only effectively extends the field of view (FOV) of the network but also ensures that the size of the feature map remains unchanged. To achieve this, extended convolution focuses on an application that can effectively integrate more background information into a broader view of the input. For the density prediction problem, Yu and Koltun et al. proposed a new extended convolution-based method for fusing data on multiple scales to improve current semantic segmentation methods. An et al. (39) proposed a “deep lab” system using “anomalous convolution” to control the resolution of feature maps in CNNs, and a new technique to segment semantic images in PASCALVOC2012 [14].

From an intuitive point of view, SRCNN [8] has a perceptual range of 13 × 13 and achieves an exciting reconstruction accuracy. The “DeepLab” system uses multiple expansion rates to extend the FOV of the network and further improve the task of semantic segmentation. Due to the results of extended convolutional techniques in semantic segmentation, a new extended convolutional neural network (DCNN) is proposed in this paper for obstacle recognition. Although the learning prior process is time-consuming, the obvious advantages of this approach are that it performs only one operation offline and can be efficiently trained in SR systems.

### 2.3. Theory of the ROI Extraction Algorithm

ROI is the abbreviation of region of interest, which means “region of interest,” and it was first proposed by the robot in the process of studying the object. The ROI zone, which is the center of human visual attention, is the most attractive and important area of research content [15].

Since the concept of ROI was introduced, ROI-related algorithms have been widely used, and various ROI algorithms have been studied to separate the object of attention from the redundant and useless background and to focus the research on the target object. After removing the irrelevant background information [16], only the ROI region is required, which can both reduce the computation and effectively improve the efficiency of image processing, as well as eliminate the influence of non-ROI regions on the system, thus improving the real-time and accuracy of the system [17].

Researchers do not select ROIs according to certain criteria but rather according to the basic algorithm of ROI, which is due to the subjective and uncertainty of ROI.

The selection of ROI has a subjective element. There is no uniform definition of ROI (ROI) in academia, and it is also difficult to perform model calculations. Also, people have different visual and aesthetic cognitive mechanisms, and when people view the same image, the focus of attention is limited by subjective will, so the delineation of ROI is determined by the user's own perception and is subjective. If a researcher can find several representative features or small objects in a photograph, the photograph can be considered worthy of study and ROI can be extracted from it. However, there are also images with generalized ROI-free regions, such as noise images, which have no value to everyone and from which ROI cannot be extracted [18].

The choice of ROI is uncertain. When selecting ROIs, regular graphics such as positive polygons, square polygons, and rectangles can be used for ROI acquisition, while non-regular graphics and custom graphics can also be used for intercepting ROIs. But to match the visual habits of users and to make the operation as simple as possible, regular graphics are generally used for ROI acquisition.

## 3. Application Method Design

### 3.1. Overall Scheme of Obstacle Detection and Type Recognition

**Figure 4:**
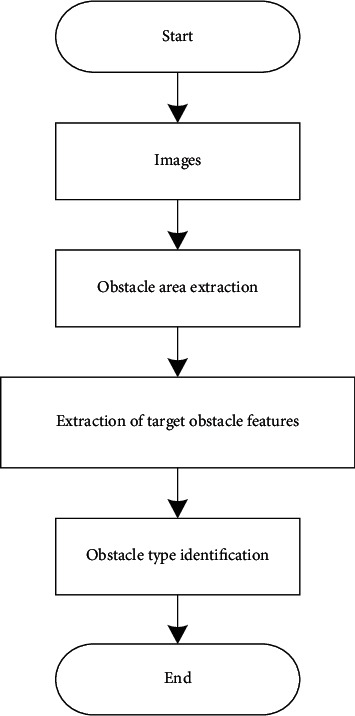
General scheme of obstacle detection and recognition.

In this paper, we introduce a method based on the widest difference method and morphological operations, the overall scheme is shown in Figure 4, firstly, the extraction of obstacles, and then on this basis, the classification of the target using the widest difference method and morphological operations; the second part is to use DCNN technology to extract the characteristics of the target obstacles, establish a kind of deep convolutional neural network suitable for multiple types of obstacles in the daily environment and combine the RPN network with the RPN network combined with it [19], the feature extraction of the target object and the recommendation of the target area are completed; the third stage is the obstacle recognition, in which the detection of multiple types of obstacles based on the DCNN network is achieved in real-time and the correct recognition results are outputted and calibrated using rectangular boxes in the actual vehicle driving. In particular, it is important to note that the accuracy of obstacle recognition described in the paper refers to the recognition probability of each type of obstacle output by the output layer, that is, the relationship between the properties of the obstacle and the real properties of the target by the artificial neural network [20].

In terms of obstacle feature extraction, the expanded convolutional neural network mainly accomplishes the following tasks:

1. Extraction of features: the principle of feature extraction is that it can describe the features of the object in the real environment. Thus, the extracted features have the following characteristics: a generic feature that can be used for the same objects, that is, they can represent the same objects; it is the differential feature that distinguishes various objects, that is, the significant difference between the characteristics of such objects and other objects; it is a robust method of describing target objects, that is, the extracted features are highly resistant to interference and can effectively avoid noise and perform target recognition.
2. By category: target object detection and recognition is based on feature extraction, using object features for classification training and determining the location of obstacles by edge regression to obtain the classification probability and specific location of the object. In this paper, we use the convolutional layer and the pooling layer to extract the target obstacles in layers and obtain the output of positive transmission through the influence of multi-layer network layers; then, the actual value and the expected value are differentiated, and a cost function is set so that the cost function tends to 0. Then, the network is updated once in the reverse direction, so that the network can be maximally close to the expected, and after repeated iterations, finally obtains with good robustness; by inputting the vehicle video into the test sample, the obstacle can be detected and recognized with high accuracy.

### 3.2. Dilated Convolutional Image Super-Resolution Design

The image super-resolution technique mainly uses IR technology to reconstruct low-resolution images. Here, ILR reduces the resolution of the corresponding HR image IHR. It should be noted that the estimated ISR is in the same dimension as the corresponding IHR and is expected to be highly similar to it. ILR is generated from the IHR, which is obtained by applying a Gaussian filter to the IHR and then downsampling the image by a downsampling r-factor. In general, the image may have C color channels, and thus the ILR can be described as a real-valued tensor of size *H* × *W* × C, and the IHR as *rH* × *rW* × *C* [21].

For the super-resolution problem of images, this paper proposes a 7-level extended convolutional neural network (DCNN), which is interpolated by an insertional ILR. Each layer can be described for the proposed DCNN as follows:

(1) $$\begin{array}{c} f^{1}\left(W_{1},b_{1}\right)=\sigma \left(W_{1}*I^{LR}+b_{1}\right)\text{,}\\ \begin{array}{c} f^{l}\left(W_{l},b_{l}\right)=\sigma \left(W_{l}*f^{l-1}\left(W_{l-1},b_{l-1}\right)+b_{l}\right),\\ f^{5}\left(W_{5},b_{5}\right)=\sigma \left(W_{5}*\left(f^{2}\left(W_{2},b_{2}\right)\cup f^{4}\left(W_{4},b_{4}\right)\right)+b_{5}\right),\\ f^{6}\left(W_{6},b_{6}\right)=\sigma \left(W_{6}*\left(f^{1}\left(W_{1},b_{1}\right)\cup f^{5}\left(W_{5},b_{5}\right)\right)+b_{6}\right). \end{array} \end{array}$$

In equation (1), *l* [2–4, 7], *i* = {*Wi*, *bi*} are the learnable network weights and offsets, where *Wi* is the weight of the *i*th convolutional layer, *bi* is the deviation term of each layer, and *i* is [1–7]. The output of layer *i* in DCNN is *fi* (*Wi*, *bi*). The weighted Wi is the 2D convolution tensor with *ni* − 1 × *ni* × *ki* × *ki*, where *ni* is the number of feature maps in layer *i*, *ki* is the filter size of layer *i*, *n*0 = *C*, and *bi* is the vector with *ni*.

**Figure 5:**
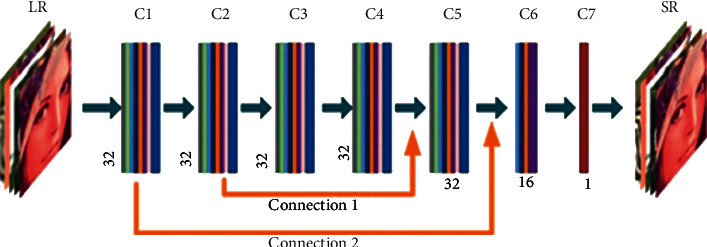
Dilated convolutional neural network (DCNN).

Figure 5 proposed dilated convolutional neural network (DCNN), which consists of seven convolutional layers with different dilation rates and two jump connections pointed by yellow arrows.

### 3.3. Image Preprocessing

The currently and commonly used obstacle detection methods are based on machine vision. Obstacle recognition technology based on machine vision has become a hot topic in the field of pattern recognition at present because of its advantages such as large amount of information and large detection distance.

**Figure 6:**
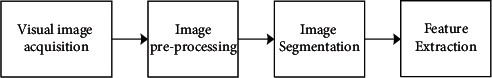
Flow chart of obstacle detection.

According to the classification of visual characteristics, it can be divided into three categories: monocular vision, binocular vision, and multiocular vision. Binocular vision, also known as stereo vision, can detect the depth and distance of the target, so that the stereo information of the target can be obtained. However, the obstacle recognition algorithm based on binocular vision has defects such as high computational complexity and easy ambiguity in the case of two small visual perception areas; while the detection method of monocular vision is easier to implement, which can be achieved by the two-dimensional information obtained by sensors such as cameras, such as shape, color, and texture,, and then the detection of obstacles is achieved by separating the object from the background region through an image segmentation algorithm. In this paper, a vision-based method is applied to detect and identify obstacles, and the corresponding preprocessing and segmentation steps are given. The flow chart for the detection is shown in Figure 6.

First, a monocular vision sensor is used to acquire visual images, which is a variety of sensors (sonar, laser scanner, line and surface array, COMS camera, CCD camera, digital camera, etc.) to acquire and collect information from the surrounding environment and convert it into a variety of electronic signals that can be recognized by the machine vision system. This thesis focuses on the acquisition of video images using COMS cameras. Secondly, the acquired images are preprocessed accordingly, and the original images are filtered to remove noise such as Gaussian and pretzel while retaining important information, and the contours, edges, and textures in the images are enhanced or emphasized. Finally, by segmenting the target region (obstacle) and background, the target region is extracted and the irrelevant background is eliminated.

### 3.4. Obstacle Region Extraction

**Figure 7:**
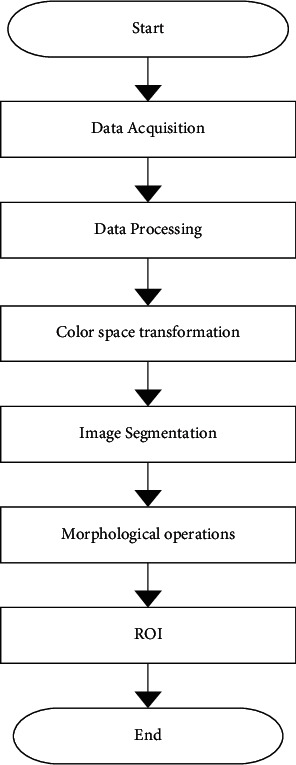
Flow chart of ROI extraction.

The flow of the algorithm based on the maximum inter-class variance method and combined with morphological operations to automatically extract ROI regions is shown in Figure 7. The specific implementation process of this algorithm is as follows: firstly, the RGB color information of the image is extracted and the components of R, G, and B color system are extracted; then, using makecform and applycform functions, the RGB color space is converted into Lab color space; based on this, the maximum variance method is used to segment the image, and according to the grey scale characteristics of the image, the graythresh function to divide the image; use the extended functions imdilate and imerode to binarise the binarised image in order to eliminate the tiny redundant targets, fill the tiny holes between regions and maintain the integrity of the ROI region as much as possible; use the fill operation to fill the gaps in the ROI region formed by the boundaries, which can be achieved by the function imfill; to remove the background, to maintain the colored information in the ROI area, to maintain the colored information in the ROI area, and to separate the ROI area from the ROI.

## 4. Experimental Analysis of Application Practice

### 4.1. Experimental Environment

In this paper, the algorithm is developed on the Windows platform using Matlab2016a, and the experimental environment and the structure of the system are given. Caffe (Caffe) is a general deep learning framework developed by BVLC (Berkeley Vision and Learning Center). The new model can be defined in textual form and its code and model can be opened up for secondary development by researchers in a specific design implementation phase, and it is fast and easy to run. A high-performance discrete graphics card of NVIDIAGeForceGTX980 Ti is chosen for better graphics processing (see Table 1).

### 4.2. Experimental Process

The whole experimental procedure is as follows.

The first step is to extract the obstacle. On this basis, the ROI region is detected using the maximum variance method and morphological operations, the ROI region is localized using rectangular boundaries, the ROI object region is separated from the original image, and an obstacle dataset containing 20,000 images is generated.

The second step is feature extraction. The images from the obstacle dataset, PASCALVOC 2007 and 2012 databases are applied to the learning and testing of the extended convolutional neural network, and five-level convolution and pooling operations are used to extract the obstacle features; the RPN network and the convolutional neural network together form a convolutional layer, thus providing the network with the obstacle area; ROI pooling is the target recognition and the region recommendation feature map of size uniformly, which is then added to the subsequent fully-connected and fully-convolutional layers for training to complete a comprehensive extraction of image features.

The third step is to identify obstacles. Five types of obstacles are designed and identified in real-time by BoundingBox, i.e., “Detections” and “Scores.”

### 4.3. Detection Results and Analysis

The proposed multiple types of obstacles were examined by the video of the vehicle measured in the field. In addition, the specific implementation of the software uses the original video as the test sample, but in order to better reflect the results of the test, this paper tests the video in frames and uses a single frame image as an example.

**Figure 8:**
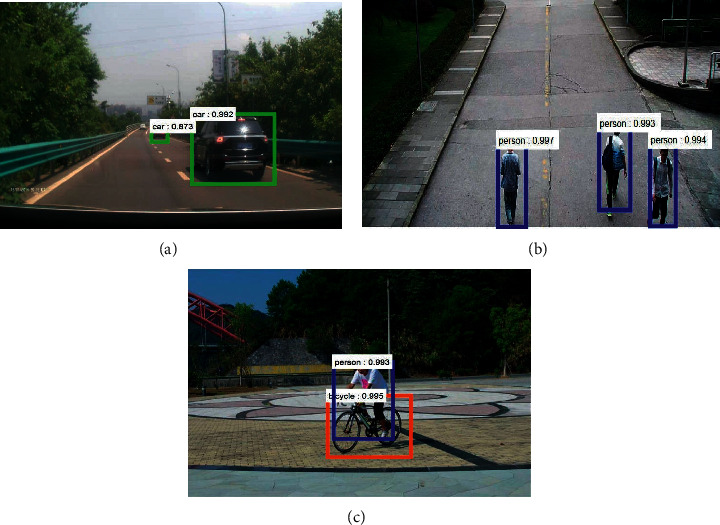
Obstacle detection and recognition under normal light simple traffic condition. (a) Vehicle inspection. (b) Pedestrian detection. (c) Human-vehicle interaction detection.

Figure 8 shows the detection and recognition of obstacles in simple traffic conditions under normal light, and it can be seen that the CNN network can detect cars and pedestrians more accurately under normal light conditions. The comparison between the 99.2% accuracy in the case of proximity and the 87.3% accuracy in the case of further distance, shown in Figure 8(a), and shows that the recognition is more accurate in the proximity of the current vehicle than in the distance, with a higher chance of recognition in general. In Figures 8(b) and 8(c), the recognition accuracy for both pedestrians and bicycles is greater than 99.3%, and in general, the DCNN network has higher than 99.3% recognition accuracy for all kinds of obstacles under general lighting conditions.

**Figure 9:**
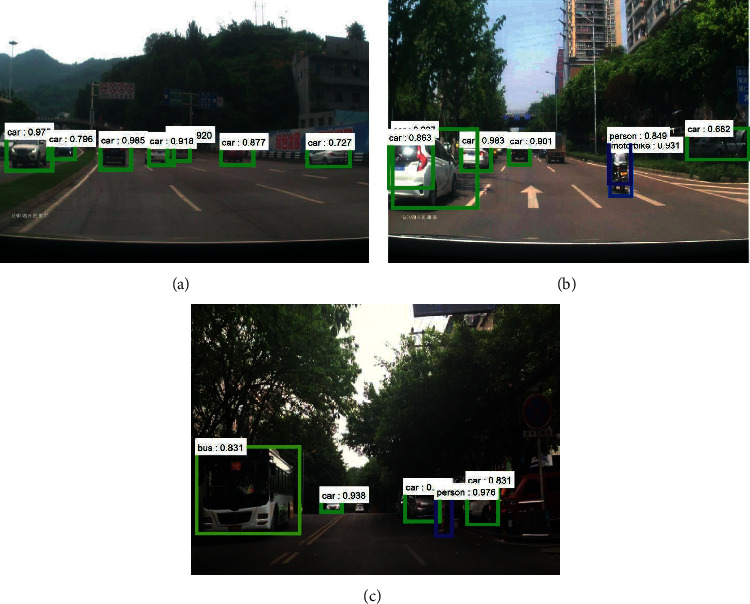
Obstacle detection and recognition under normal light complex traffic conditions. (a) A wide range of vehicles in the driving environment. (b) Human-vehicle interaction in the driving environment. (c) Human-vehicle interaction in the driving environment.

Figure 9 shows obstacle detection and recognition in complex road conditions under normal light conditions. From this figure, it can be seen that when there are various obstacles in the vehicle driving environment, such as cars, people, motorcycles, and buses, the deep convolutional neural network established by using this method can detect the obstacles in the vehicle driving environment with a high recognition rate. With incomplete obstacle information, it can be detected accurately, for example, in Figure 9(a), when there are a large number of vehicles, the DCNN network can identify both the front and side obstacles of the car. For the interaction behaviors in traffic environments such as cars, people, and motorcycles, the DCNN network is able to detect and identify various obstacles with an accuracy of over 60% for all of them and 90% probability for most of them.

The detection of each frame of obstacles performed by the DCNN network used in this thesis is comparable to the time required for the classification of PASCAL VOC, which is around 0.055 seconds, ensuring real-time detection. In general, the algorithm is able to accurately detect and identify various obstacles in real-time with high accuracy, both in simple traffic environments and in complex road conditions.

To verify the effect of this method on obstacle detection under normal light conditions and road conditions, a total of 35,500 detection frames were collected, in which the number of detected obstacles reached 95,920, and the related detection results are shown in Table 2. From Table 2, it can be seen that the accuracy of obstacle recognition with the *D* CNN network under normal light conditions reached 92%, and under the dim environment, its accuracy reached 86%. At the same time, its recognition is better in simple road conditions than in complex situations.

The reliability of the DCNN network with good universality and robustness is verified by obstacle detection under various road conditions. The method can effectively solve the technical requirements of conventional obstacle detection methods in terms of accuracy, reliability, and universality that cannot be adapted to the fields of smart cars and UAVs. Moreover, the test device of this topic has a lower cost compared with the conventional test equipment, which has a great practical value.

## 5. Conclusion

Obstacle recognition and type recognition are currently the most basic and challenging topics in the field of computer vision, and in China, with the increasing number of cars and the continuous development of smart cars and driverless technology, road traffic safety has become a top priority. Using the weight-sharing hierarchical structure of the expanded convolutional neural network, we classify all kinds of obstacles and extract representative overall features from them and combine the ROI method with it to achieve high accuracy classification recognition. The shortcomings of traditional obstacle detection and recognition methods in terms of accuracy, reliability, and universality, as well as the high cost of traditional detection equipment, are addressed.

## Figures and Tables

**Table 1:** Experimental environment and configuration.

Experimental environment	Environment configuration
Operating system	Windows
CPU	8 nuclear InterI CoreI i7-6700K CPU @ 4.00 GHz
Memory	16 GB
Video card	NVIDIA GeForce GTX 980 Ti
Programming language	Matlab2016a
Database	PASCAL 2007, PASCAL2012
Deep learning framework	Caffe

**Table 2:** Statistics of detection results under different light and traffic conditions.

Light conditions and traffic conditions	Detection of frames	Number of obstacles	Average recognition accuracy (%)
Normal light simple traffic conditions	12000	15020	95.1
Normal light complex traffic conditions	8000	33840	92.1

## Data Availability

The dataset used in this paper is available from the corresponding author upon request.
